# “Intelligence Running Wild”: Edward Podvoll (1936–2003) and the Unfolding of the “Contemplative Psychotherapy” Project

**DOI:** 10.1002/jhbs.70034

**Published:** 2025-08-18

**Authors:** Tommaso Priviero

**Affiliations:** ^1^ Leverhulme Early Career Fellow Queen Mary University of London (QMUL) London UK

**Keywords:** buddhism, history of psychology, meditation, psychoanalysis, psychotherapy

## Abstract

This paper explores the origins and development of the “contemplative psychotherapy” project in the United States, emerging around psychoanalyst Edward Podvoll and the intellectual environment at Naropa University during the 1970s and 1980s. It situates Podvoll's work within the broader dialogue between Buddhism and Western psychotherapy, highlighting how his contributions shed light on this dynamic and complex encounter. The paper begins by outlining Podvoll's psychiatric and psychoanalytic background, then examines the establishment of contemplative psychotherapy at Naropa, and finally discusses the creation of the Windhorse community therapy model—while contextualising Podvoll's dialogues with influential figures such as Chögyam Trungpa, Erik Erikson, Manfred Bleuler, and Oliver Sacks. Drawing on published and unpublished material by Podvoll, it argues that his work exemplified a pioneering effort to integrate Buddhist and psychoanalytic perspectives on mental health and recovery. At the same time, the paper reflects on some of the conceptual and therapeutic tensions that arise when spiritual and clinical paradigms converge. By examining contemplative psychotherapy as both a historical case study and a continuing experiment, the paper contributes to ongoing debates about the possibilities—and limits—of East–West intersections in psychological practice.

## 1 Introduction

Towards the end of his life, the British historian Arnold Toynbee claimed that the arrival of Buddhism in the West would be one of the most impactful events of the 20th century (Keown 1996, 122). Decades later, there are many facets of this mercurial event that have yet to be understood. However, there is little doubt that the encounter between Buddhism and Western psychology in the 20th century has emerged as a highly significant and complex issue in the history of the human sciences. As the Zen Buddhist priest and clinical psyhcologist Seth Zuihō Segall noted, for over a century “psychologists, psychiatrists, and psychoanalysts have analysed, pathologized, misinterpreted, appreciated, assimilated, adapted, and/or converted to Buddhist ideas and practices” (Segall 2024, 1). Segall identifies a variety of reasons to explain this process: the idea that compared to other religions, Buddhism would be the most compatible with Western science (Cabezón 2003; Lopez 2009, 2012), the common concerns arounds topics such as the nature of the self, mind, and altered states of consciousness, the central role of meditation, as well as the international appeal of Tenzin Gyatso, the 14th Dalai Lama (Segall 2024, 5–6). Spanning over multiple decades, historians have started to address the “psychologisation” of Buddhism in Western culture as a widely heterogenous process which has persistently impacted psychological disciplines in ways that keep evolving. Since the beginning of the 21st century, different historiographic trajectories have emerged within this field. In a seminal work, Lopez (2002) introduced the concept of “modern Buddhism”, referring to the process that gave rise to an international Buddhism that transcends cultural and national boundaries, creating “a cosmopolitan network of intellectuals, writing most often in English” (2002, xli). Following a similar direction, McMahan (2008) focused on “Buddhist modernism”, exploring those forms of Buddhism that emerged out of an engagement with the “cultural and intellectual forces of modernity” (2008, 6) and shared a few key traits such as demythologisation, a scientific orientation and a central emphasis on meditation. Meanwhile, Segall (2003) provided one of the first detailed accounts of personal and professional encounters between Western psychologists and Buddhist teachings. Similarly, Dockett et al. (2003) adopted an interdisciplinary approach to bridge Buddhist and psychological perspectives, emphasising ethical and community‐oriented frameworks as integral to both traditions. Other scholarly works have examined the transmission of Buddhism through influential figures and practices, such as John Teasdale (Drage 2018) and Carl Gustav Jung (Knox 2021, 2025). Goble's history of Buddhism (2019) challenged misconceptions and false beliefs in the Western reception of historical Buddhism, navigating the space between “facts” and “fictions”. Finally, more recent contributions (Cohen 2010, 2022; Segall 2024) have continued to critically assess how Buddhism has been reconstructed in the modern West, primarily through scientific and psychological idioms.

Against this background, this paper focuses on a hitherto overlooked chapter of the reception of Buddhism in the history of psychology: the “contemplative psychotherapy” project, which arose around Edward Podvoll (1936–2003) and the intellectual milieu of Naropa University (Boulder, Colorado), in the early 1970s. The term contemplative psychotherapy defined an attempt to bridge the science of Western psychology with Buddhist philosophy. This was a topic of interest for some of the early founders of modern psychology, such as William James and C.G. Jung, as well as intellectuals such as Alan Watts and Travers Christmas Humphreys. Jung, in particular, was the first psychologist to attempt to frame Buddhism and Eastern religion in psychological terms, by juxtaposing Buddhist ideas to his own analytic notions, thus pioneering the modern reconfiguration of Buddhism as a psychological science. However, Jung also noticeably warned Westerners against uncritically adopting Eastern meditation practices, believing that the West could have developed its own “yoga tradition” through alchemy and the evolution of Christianity. Differently, the early 1970s saw the transformation of Buddhism from an object of intellectual fascination to a living practice within Western psychotherapeutic traditions. In this respect, the contemplative psychotherapy project at Naropa was arguably the first historical attempt at proposing a psychotherapy model tout court in which the insights of Buddhism and Western science could coexist. Podvoll introduced the term contemplative psychotherapy when the first department for the study of such discipline was created at Naropa, as well as a *Journal of Contemplative Psychotherapy*, which ran from 1980 to 1994. Wegela, a contemplative psychotherapist who is still active at Naropa today, defined contemplative psychotherapy as “the child of two different parents”, which “just like all offspring, […] has much in common with both its parents, but it has grown up to become something different from either one” (Wegela 2014, 1). In Podvoll's own words, contemplative psychotherapy referred to the “quality of treatment that results from joining the interpersonal discipline of psychotherapy with the personal discipline of working with oneself through the practice of meditation” (Podvoll 1987, 1). Today, in an age where the image of the Buddha in meditation has become nearly synonymous, in the popular imagination, with psychological wellbeing and healing, the term “contemplative” is widely used across psychotherapies, the arts, and neuroscience—so much so that Loizzo, the founder of the Nalanda Institute for Contemplative Science, claimed that the framework of contemplative disciplines has outgrown the popular term “mindfulness” (Loizzo et al. 2017, xxv). However, to fully grasp the roots of this terminology in psychological contexts, it is important to understand its original and significant connection to Naropa University. Thirty years after the creation of the “contemplative psychotherapy” model, Podvoll observed: “The encounter of Buddhist meditation practice with Western psychological treatment has been gradual but continuous for the past 30 years, and the result has been both subtle and revolutionary” (Podvoll 2003, xi). This paper will probe these claims, with two main goals: (1) to position Podvoll's work within the broader context of the history of psychology, and (2) to highlight how the contemplative psychotherapy project conveyed one of the earliest attempts at integrating Western psychological science with Buddhist philosophy.

## 2 Who Was Edward Podvoll?

Edward Podvoll was born in 1936 in Brooklyn, New York, into a family of Jewish doctors. Growing up in a home that doubled as a medical practice, he was exposed early to the rhythms of care and recovery (Podvoll 2003, 306). These formative experiences later shaped his therapeutic concept of “basic attendance”—a simple, direct presence with another person, which became a cornerstone of his approach to psychotherapy and healing communities. He observed that when a child grows up accustomed with the practice of basic attendance, this creates “a warm spot and an easy acceptance for all future experiences of basic attendance” (Ibid., 349), enabling the child's openness to enter relationships with others. Jeffrey Fortuna, a long‐time friend and colleague of Podvoll, as well as a key figure in the development of Windhorse, described basic attendance as “getting down to what is immediately relevant (i.e., ‘basic’) to being with someone and doing what is required at the time (i.e., ‘attendance’)” (Fortuna 2011).

After becoming an MD in 1961, aged 25, Podvoll started working at the Chestnut Lodge Sanatorium in Rockville, Maryland. Meanwhile, he married his first wife in New York, who gave birth to his only daughter, Stacey. Chestnut Lodge was home to nationally renowned clinicians such as Frieda Fromm‐Reichmann and Harold Searles. Around that time, it was gradually transformed from a sanatorium for alcoholic recovery into an internationally known institution for psychotherapy with psychosis. It was customary for resident doctors to see at least six hospitalised patients four times a week. As many of the clinicians involved were psychoanalysts, the work at the clinic had a strongly psychoanalytic orientation. Thus, in 1963, Podvoll began a 7‐years long personal analysis with Searles, while embarking in a psychoanalytic training at the Washington Psychoanalytic Institute. Searles himself had been in training analysis with Philip Graven, who in the 1920s went to Vienna to study psychoanalysis and was analysed by Sigmund Freud. Well‐known for his eccentric style, Searles had gained an international reputation for the treatment of severe mental illness as well as for his expertise on the phenomena of transference and countertransference (Searles 1965, 1979, 1986). His work was influential, particularly among the founders of the relational school of psychoanalysis (Aron and Lieberman 2017, 183). Podvoll's encounter with Searles was pivotal, at personal and professional levels (Podvoll 2003, 328). He highlighted Searles's unequivocal attention to the experience of what psychoanalysts call “negative transference”, that is, the analyst's skills to receive and interpret the patient's uncomfortable or hidden emotions towards the therapist, including aggressiveness, resentment, disappointment, malice, envy. He noticed that Searles felt that “failure to acknowledge these feelings” in order not to cause problems to the therapeutic relationship, was perhaps “the greatest single cause of therapeutic failure, of stifled and lifeless therapy” (Ibid., 330). At the same time, he seemed to be impressed by Searles's unconventional lightness and humour in therapeutic practice, claiming that he never laughed so much with anyone else (Ibid., 332).

After working at Chestnut Lodge, he moved to Austen Riggs Centre, in Stockbridge, Massachusetts, where he met, among others, the influential psychoanalyst Erik Erikson. Coinciding with these years, he began to develop a serious interest in Buddhist philosophy and practice. This came from a mixture of readings and personal experiences, varying from explorations into altered states of consciousness through psychedelic drugs (which Podvoll became increasingly sceptical about, over the years), to meditation practices. The reading of William James, in particular, pushed him towards the study of the psychology of Eastern and Western religious experiences, which the American psychologist dissected in his masterpiece *The Variety of Religious Experience* (1902). James, along with the German‐American author Paul Carus, played a significant role in shaping the encounter between American philosophy and Asian traditions at the end of the 19th century. As David Scott suggested, while there had been earlier fascination with Buddhism among the American Transcendentalists, James's direct references to Buddhism and the thematic resonances between pragmatism and Buddhist philosophy made this engagement more explicit (Scott 2000, 335). Besides James, Chögyam Trungpa's pivotal *Cutting Through Spiritual Materialism* appeared in 1973, which became soon a classic among Westerners who gravitated to Buddhism. However, far from being just a scholarly interest, he began to engage with Buddhist practices and, starting from the autumn of 1974, he was a regular visitor to the meditation retreat center Karmê Chöling, in Vermont, where he became a student of Trungpa.

In August 1975, he gave a talk entitled “Psychosis and the Mystic Path” to the Fifth International Symposium on the Psychotherapy of Schizophrenia, in Oslo, Norway (later expanded and published in *The Psychoanalytic Review* in 1979). This paper was a turning point in his developing views around psychotic or “extreme states of mind”, as he later preferred to say, and made Searles worry that his former analysand had ‘lapsed into mysticism’.^1^ In Jamesian fashion, the talk attempted a non‐reductive interpretation of altered states of consciousness, exploring a basic common ground between madness and the mystic condition, without “idealising one or pathologizing the other” (Podvoll 1979, 571). While drawing on a few classical psychoanalytic sources, including the works of Freud and his daughter Anna Freud, the talk also explored Mircea Eliade's study of ancient shamanic techniques of ecstasy and R.G. Wasson's research into hallucinogenic mushrooms, which led to the popularisation of psilocybin compounds. “Psychosis and the Mystic Path” revealed Podvoll's multiform intellectual pursuits. Around this time, these interests began to flourish through the mediation of Tibetan Buddhism, which appeared to offer practitioners a phenomenology of non‐ordinary mental states as well as techniques to explore them directly. When these insights became embodied by the charismatic appeal of Trungpa's meditation teachings, they brought a shock to Podvoll's system. Years later, he recalled of this moment:

When trained psychotherapists began to experience the deeply personal insights of their meditation practice, it changed their lives. What they were learning turned Western psychology upside down, because they were exposed to a whole new way of seeing mental suffering and mental healing. […] In 1974 I became one of those therapists. Although I had completed extensive training in psychoanalysis and was already a teacher of that discipline, what I was discovering with the practice of meditation made it impossible for me simply to continue that way.

(Podvoll 2003, xi)

In 1976, Trungpa invited Podvoll to move to Boulder, Colorado, where 2 years before he had founded the Naropa Institute, which later became the first Buddhist‐inspired University in the Western world. The doctor and psychoanalyst from New York, then aged 40, agreed without further ado.

## 3 Naropa and the Contemplative Psychotherapy Project

Psychology and Buddhism, though both relatively recent areas of academic inquiry, emerged from independent origins. Over time, however, their histories began to intersect. Western scholarly interest in Buddhism has a particularly complex background, rooted in 19th‐century developments in philology and shaped by colonial agendas—as well as the postcolonial critiques that followed. Psychology, meanwhile, evolved from a diverse array of pre‐1850 movements and disciplines, including mesmerism, hypnotism, physiology, phrenology, ethology, philosophy, and theology. Despite their autonomous origins, both Buddhism and psychology came to be seen as responses to the spiritual discontent that marked the 19th and 20th centuries. Their encounter unfolded through several distinct phases. In the early 20th century, Buddhism attracted the attention of Orientalist scholars and theosophical circles, particularly in England. Later, with the rise of psychoanalysis (especially through Jung) it was reinterpreted through the model of the “unconscious” mind. By the latter half of the 20th century, this engagement had evolved into an active integration of Buddhist ideas within Western psychotherapeutic practices. In this context, newly established institutions such as Naropa University, along with the arrival of Eastern teachers into Western cultural spaces, were instrumental in shaping a different dimension of this dialogue.

The Tibetan teacher Trungpa is appraised as one of the pivotal figures in the spread of Buddhism in the West in the 20th century. Born in Tibet in 1939, he was recognised as a *tulku* in his childhood, that is, in Tibetan Buddhism, the reincarnation of a deceased Buddhist master of a high spiritual lineage. Upon fleeing Tibet following the Chinese occupation of the Land of Snows in 1959, he moved to Oxford, England, and then to Scotland, where in 1967 he co‐founded with Akong Rinpoche the Kagyu Samye Ling Monastery (the first Tibetan Buddhist Centre in the West), near the small village of Eskdalemuir. In January 1970, he married Diana J. Mukpo (née Pybus) and moved with her to North America, founding a great number of meditation centres across the United States and worldwide, while settling in Boulder. Trungpa was widely regarded as a brilliant yet controversial teacher. In sharp contrast to traditional Buddhist norms, he was known for his extravagant lifestyle, which included heavy drinking (he died at 48 from complications related to alcoholism), sexual relationships with some of his students, and the use of provocative, often shocking teaching methods to spark insight. While this partly reflected the broader context of the 1970s counter‐cultural movement, which rejected conventional moral frameworks, Trungpa's actions also raised serious ethical concerns and had troubling consequences. A kind of cult personality developed around him, attracting several criticisms, including allegations of manipulation and reports of personal crises among some of his disciples. Despite this, he gained widespread recognition for his skilful ability to make Buddhist teachings accessible to Western audiences. Coming into contact with Western culture convinced him of adopting Western dress and manners, as he envisioned that Buddhism would land into the West as a psychology, rather than a philosophical or religious tradition. As Gimian pointed out, Trungpa's use of themes such as “doubt, trust, depression, anxiety, and neurosis” was highly unconventional for Buddhism at this point in time:

His use of psychological terminology and themes may well be viewed, in the long run, as one of his major contributions to the development of Buddhism in the West. Psychological vocabulary as a vehicle to express the deepest truths of Buddhism in now commonplace, taken for granted by readers and practitioners. But it was anything but the norm at the time that Rinpoche went to England and then journeyed on to America.

(Gimian 2002, xviii)

Anticipating a process which many years later scholars would refer to as the “psychologisation” of Buddhism (Lopez 2009, 2012), Trungpa's characterisation of Buddhism as a new “psychology” in the West offered a provocative starting point for what would later become a broader historical trajectory—although one not without tensions, especially regarding the risk of reducing complex systems to therapeutic tools. The choice of a psychological vocabulary invariably attracted professional psychotherapists and psychologists, such as Podvoll, who were interested in broadening the horizons of classical therapeutic disciplines. Similarly, Trungpa was interested in engaging with the practice of psychotherapy and, as early as 1971, he began to put together plans for therapeutic communities to work with suffering individuals. He was convinced that a limitation of Western psychotherapy was that it emphasised theory much more than “experience” (Trungpa 2005, 4–5). By “experience”, he meant firsthand observation of one's mind, as developed through the practice of sitting meditation. Furthermore, he regarded his encounter with the Western religious notion of “original sin” as a shock, which, he argued, had been inherited by psychology with its excessive focus on an individual's sense of being intrinsically “wrong” or “wounded” (Ibid., 8–9). He counterposed to that the Buddhist idea of “basic goodness” or “brilliant sanity”, that is, a fundamental disposition to health and wakefulness which existed in every individual. He warned against taking this notion naively, as a “goody‐goody principle”, defining it rather as a non‐dual state of mind that takes place “before even the notion of good and bad happens at all” (Trungpa 2005, 81). In an article entitled “Becoming A Full Human Being” (1980), he singled out a therapeutic model based on a radical form of compassion, openness, long‐term commitment, and mutual appreciation between patients and practitioners. “The ability to work with another person's neurosis, or even their craziness”, he wrote, “ultimately depends on how fearless you are when you deal with them, or how inhibited you feel” (Trungpa 1980, 141–219). Overall, Trungpa found the meeting of Buddhism and Western psychological disciplines of great importance. A clear sign of this was that, as soon as Naropa institute opened in 1974, a master's degree in contemplative psychology was developed with the intent to train clinical psychologists with an interest in Buddhism.

According to Lief, one of Trungpa's senior editors, Podvoll was a “pivotal figure in the development of this program” (Gimian 2002, xxii, n10), which later became the Department of Contemplative Psychotherapy. The Department promoted training in meditation practices, courses and seminars for the study of Buddhism and Western psychology, as well as internships in therapeutic settings. Naropa became a kind of pilgrimage destination for Western students of Eastern philosophy, including intellectuals, artists, and contemporary counter‐cultural figures, such as G. Bateson, R.D. Laing, A. Ginsberg, A. Waldman, J. Cage, and many others. Around 1977, Podvoll became director of the Department and maintained this role for the next 12 years. During this time, he taught seminars and lectures, the recordings of which are largely preserved in the extensive Podvoll archives of the Windhorse Legacy Project. The subjects of these seminars varied from dreams and therapeutic practice to the history of psychology and psychopathology. Only a small part of this material (a three‐seminar series taught at Naropa in 1985–1986) has recently been published in digital format by the Windhorse network with the title *Healing Discipline*, signalling fresh interest in the rediscovery of Podvoll's works, which for the most part awaits editing and publishing deliberations.

In 1978, Daniel Montgomery and Fortuna, both active at Naropa, founded the *Journal of Contemplative Psychotherapy* (until 1985 known as the *Naropa Institute Journal of Psychology*). Podvoll agreed to become the Editor‐in‐Chief of the *Journal*, which in the next 14 years published nine issues and was at the front line of the Western fascination with the East, anticipating many of the themes which the “mindfulness movement” pursued over the next several decades. As reported in a personal conversation with Gimian, Podvoll seemed to be aware of the future potential of the encounter of Buddhism and Western psychology, referring to it as “something of a cultural explosion” (Gimian 2002, xxiii). In a preface to the first volume of the *Journal*, entitled “Brilliant Sanity”, further emphasis was placed on an “inherent state of wakefulness that can be pointed to, recognised, and encouraged through psychological work”; this was not intended as a metaphysical principle, but as a simple and direct experience arising from “clarifying the nature of mind processes” through “discipline, gentleness, and courage” in working with oneself and others (Podvoll et al. 1980, 1). Interestingly, the preface also listed Darwin, William James, Freud, and Lacan as key contributors to the study of the origins of self‐consciousness and the problems of its definition. Over the years, the journal received regular submissions from figures coming from different backgrounds—the Vietnamese Buddhist monk Thich Nhat Hahn, the Scottish psychiatrist R.D. Laing, the British neurologist and historian of science Oliver Sacks, the pioneer of the notion of therapeutic community Maxwell Jones, the psychoanalysts Mark Epstein and Farrell Silverberg, the scholars Judith Simmer‐Brown, Han de Wit, and Karen K. Wegela, among others. The material comprised clinical studies and reflections on environmental treatments, with a keen eye on the points of encounter and divergence between Buddhism and psychology.

At Naropa, academic research was closely tied to practical applications, whether these occurred through meditation exercises or community interventions. In a similar spirit, in 1981, Podvoll gave birth to the Windhorse project. The term “wind‐horse” (*lungta*) in Tibetan Buddhism designates a mythic flying horse that represents “life force” and often appears in Tibetan prayer flags and statues. As Fortuna pointed out, “Wind‐horse began as an outgrowth of our training with Chögyam Trungpa Rinpoche at Naropa University […], who inspired us with the vision of intrinsic health and gave us the means to create healing environments” (Fortuna 2003, xiii‐ix). The Windhorse project was inspired by the objective of creating home and community‐based clinical teams for individuals who were recovering from extreme mental states, often after periods of psychiatric hospitalisation. From the mid‐1960s to the mid‐1980s, the idea of experimental healing communities had been flowering across Europe and America, with examples as various Kingsley Hall (R.D. Laing), Burghölzli Hospital (M. Bleuler), Chestnut Lodge (H. Searles), Dingleton Hospital (M. Jones), Diabasis House (J.W. Perry). In a similar counter‐cultural spirit, Windhorse shared with these projects a desire to offer alternative healing modalities to orthodox biomedical paradigms. However, unlike some of the more radical anti‐psychiatric communities of the era, Windhorse oriented itself more towards contemplative frameworks than political antagonism. It was guided by the belief that healing arises not only through social reintegration, but also through cultivating environments that support meditative insight and practices at the core of a person's life. Windhorse patients lived in households with a housemate who was part of the treatment team, receiving tailor made individual treatment while engaging in everyday life activities.

When Podvoll informed Manfred Bleuler (Eugen Bleuler's son) of his attempts at creating Windhorse communities, the Swiss psychiatrist responded that “he and his father had always ‘dreamed’ of creating such treatment teams at the Burghölzli Hospital in Zurich, but many hundreds of admissions a year made it impossible” (Podvoll 2003, 216). Windhorse treatment plans were created following the principle of taking care of patients in the environment that felt most natural to them, while tending to their most immediate needs (“basic attendance”), which often were domestic in nature (Ibid., 248). Podvoll recounted an example of this process in *Recovering Sanity*, when describing a patient who, after having done very well in recovery, entered an acute crisis, becoming completely fixed in a hallucinatory world. The team leader (Fortuna) called Podvoll for support and he went over to the patient's home, where the patient was huddled in the corner of his bed, totally unresponsive. Any attempt to talk with him and get him out of his fixation appeared useless, until Podvoll and Fortuna gave up, feeling helpless, and started talking to each other of personal matters, as two friends would do. An unexpected change occurred then:

Somehow we got quite involved talking to each other […] and we were thoroughly enjoying our conversation, and sometimes laughing. What a relief from the intensity of wanting something from the patient! […] Gradually the patient attended to us. Maybe he found our world more interesting than the one he was stuck in, or he was curious about our lives, or simply jealous, who knows. Little by little, without the least attempt on our part to seduce him, he began to join our conversation. This was not any particular triumph on our part, but it was for the patient an unusual experience of being able to break loose from his thought‐world without having to be outrageous or violent.

(Podvoll 2003, 324)

When the first therapeutic household was created for a patient named Karen, in 1981, a primary school teacher from Los Angeles called Candace was invited to be her housemate, together with her daughter and sister. On this occasion, the arrangement only lasted for 6 months, as it was drawn to an end at Karen's request, who remained in individual psychotherapy with Podvoll. Karen's case was outlined in *Recovering Sanity* and in Edgar Hagen's movie *Someone Beside You* (2007). She became a protagonist in the story of Windhorse, as an emblematic case of the Windhorse approach. In January 1999, after receiving a card from Karen, Podvoll wrote to Fortuna: “I was very moved and I wrote her a letter. Of course, she changed my life as much as I did hers”.^2^ Meanwhile, a romantic relationship developed between Podvoll and Candace. Eventually the couple got married in 1986 and remained together until his death. Podvoll's independent and complicated character, as well as his commitment to lifelong retreat from 1991, did not make the marriage a conventional one. Nonetheless, the couple were united by a mutual devotion to Buddhist practices (once Podvoll joked that Candace “may be even more of a religious fanatic than I am”),^3^ traveled to Europe and India together, and shared affection over the years, as revealed by the letters which they continued to send each other even at a distance.

**Towards** ***Recovering Sanity***

While the objective of the contemplative psychotherapy project was to reconcile Western scientific language with the insights of Buddhism, the focus on meditation practice became the place par excellence where this encounter could take place. The use of meditation within the history of Western psychotherapy and psychoanalysis is a complex and overlooked subject, which goes beyond the scopes of the present paper, but calls for future investigation. As Rubin noted first (1985, 599), Freud's notion of the analyst's evenly suspended attention (a receptive form of listening that mirrors the patient's free associations), showed affinities with aspects of the Buddhist practice of bare attention—a state of mind in which phenomena are observed without judgment or mental elaborations. Later, the British psychoanalyst Wilfred Bion proposed the model of the analyst listening “without memory, desire and understanding” (1970), strengthening this link further. However, while the Freudian tradition generally maintained a critical stance towards spiritually connected practices, Jung and Jungian psychoanalysis went a step further, introducing the technique of “active imagination” (a mental state akin to a “dream with open eyes”), a practice that involved an intentional dialogue with “unconscious” contents (Jung 1916/1957), often through visualisation or symbolic interaction, with the psychotherapist helping the analysand integrate emerging images. However, where “active imagination” invited imaginative elaboration, Buddhist meditative traditions often regarded elaboration itself as a source of delusion, even though both approaches aimed at a transformation of the subject's relation to their inner life. As Marco Innamorati pointed out, the relationship between psychoanalysis and meditation evolved in the course of time. Initially, meditation was regarded as a subject for theoretical exploration rather than clinical application, with some considering its practice counterproductive or even harmful. From the 1970s onward, however, shifts in psychoanalytic thought led to the decline of Freudian metapsychology and the rise of alternative psychotherapeutic models, which opened the door to viewing meditation as a complementary tool within or alongside psychoanalytic treatment (Innamorati 2024, 16–17). This change of perspective allowed the interest of contemplative psychotherapy in Buddhist meditation to properly unfold.

As highlighted by Kalupahana (1987), among others, William James's pragmatist philosophy provided an excellent point of contact with Buddhism to a Western audience. The American psychologist, who in a postscript to *The Varieties of the Religious Experience* claimed to agree in principle with the Buddhist non‐transcendentalist “doctrine of Karma”, also noted the extraordinary importance of the faculty of attention for psychological health:

The faculty of voluntarily bringing back a wandering attention, over and over again, is the root of judgment, character and will. […] An education which would improve this faculty would be the education for therapy par excellence. But it is easier to define this ideal than to give practical direction for bringing it about.

(James 1983, 401)

From the perspective of contemplative psychotherapists, Buddhist psychology answered this call in an ideal way, by offering “practical direction” in the technique of developing attention and concentration. Wegela emphasised this aspect as follows:

In 1985, I attended a large gathering in Phoenix, Arizona, the first Evolution of Psychotherapy Conference. It brought together practitioners and students of every stripe of psychotherapy. Among the presenters were R. D. Laing, Carl Rogers, Carl Whitaker, James Bugental, Thomas Szasz, Virginia Satir, and many others. There were thousands of participants. I was struck by how often the presenters pointed to the same ability as the most important asset that a therapist could have: the ability to be present with clients. Then, just as often, they would go to say something like, ‘Too bad we can't train that.’ They seemed to think we had to be born with it. As I sat with a few of my Naropa colleagues, we turned and looked at each other in surprise. We knew that this quality could be trained, and we knew that meditation practice could train it.

(Wegela 2014, 38)

Through the 1980s, Podvoll wrote continuously until 1990 in preparation for the publication of his only major monograph, *The Seduction of Madness*. During this time, he was one of those practitioners who explored the possibilities of the healing effects of meditation. In *Someone Beside You*, he described his encounter with meditation after psychoanalytic training as an epiphany: “This is the real thing. This is how to study mind. This is how to change at a fundamental level” (46′50″−46′58″). He recalled that, following this encounter, “everything was changing. The whole idea of therapy was changing. My mind. I was working with people differently” (47′03″−47′10″). On this occasion, he borrowed the psychoanalytic term “depth psychology” (*Tiefenpsychologie*, originally coined by Eugen Bleuler in the late 19th century), to refer to Buddhism (47′32″), by contrast with more conventional psychological approaches. While meditation profoundly transformed Podvoll's outlook, he continued to practice psychoanalysis and psychotherapy, first at the University of Colorado, and later in connection with Windhorse communities. Between 1981 and 1987, he saw patients regularly, sometimes up to four times a week, and continued prescribing medications as a psychiatrist. Although there is no evidence that he directly used meditation techniques in therapy sessions, meditation deeply shaped his therapeutic attitude and played a central role in his teachings. Podvoll's encounter with meditation led his approach to contemplative psychotherapy to diverge from traditional psychoanalysis. Still, some psychoanalytic features remained central to his practice: frequent and intensive sessions, dream analysis, and a close focus on transference and countertransference. His therapeutic style also reflected the relational approach of Searles and Fromm‐Reichmann. He maintained a personal correspondence with Searles well into later life and remained attentive to his mentor's views. In a late essay entitled “Psychotherapy,” Podvoll identified Searles's “essential teachings” as a major influence on his clinical work. He also engaged extensively with Freud's writings and occasionally referred to Lacan in his seminars. Notably, he rarely mentioned Jung, who pioneered the encounter of Western psychology with Eastern philosophies. At the time, Western psychoanalysts and psychologists approached the subject of Buddhist meditation in very different ways. On the one hand, the search for a common ground was evident by publications which even rose to the status of bestsellers, such as E. Fromm, D.T. Suzuki, and R. De Martino's classic *Zen Buddhism and Psychoanalysis* (De Martino et al. 1960). A few psychoanalysts, such as Nina Coltart, began to integrate their personal Buddhist meditation practices (Theravada Buddhism, in Coltart's case) into psychoanalytic work, while holding a cautious sense of the differences and similarities between the two (Coltart 2015). On the other hand, other psychoanalytic figures maintained a more skeptical standpoint. Erikson, for example, when informed by Podvoll of his decision to bring meditation into psychoanalytic practice, told him without reservations: “What you are doing is in complete contradiction to my life's work” (Erikson, cited in Podvoll 2003, xiii).

According to Podvoll, this contradiction essentially resided in the “problem of the ego”. In his view, traditional Western psychology broadly posited that “sanity” derived from the stability of one's sense of self (broadly speaking, the “ego”) and, correspondingly, mental disturbance arose from a crisis of that security, that is, a failure to secure one's personal “territorial demands”. Conversely, contemplative psychotherapy followed the opposite principle that “sanity” came from the “gradual softening and dissolving” of one's self‐absorption, leading to internal relaxation. Thus, he referred to the “ego” as a “personal mythology” and conceptualised neurosis as the outcome of habitual patterns and anxieties that were put in place to handle one's personal hopes and fears. From this perspective, the way to cut through this illusion was found in a difficult process of befriending oneself, which primarily took place through sitting meditation. A practice which, at this point, Podvoll engaged with for at least 3 h a day:

During the first 3 years I experienced a more subtle level of mind and its movements than can ever be seen in psychoanalysis. It was below and before the level of speech and communication, far more fleeting and delicate than the most open levels of free association. […] Gradually, I understood a new dimension of mental suffering that I had only glimpsed during psychoanalysis. […] I began to understand my patients differently. No matter how disturbed they were, their basic psychological ordeal was the same as my own. I felt more openness and kindness toward them and spoke more easily and directly with them.

(Podvoll 2003, xii)

This radical shift of perspective into the study of mental phenomena became the focal point of Podvoll's research, coalescing into his book *The Seduction of Madness* (1990), later revised and retitled *Recovering Sanity: A Compassionate Approach to Understanding and Treating Psychosis* (2003). In fact, Podvoll's proposed title for the 1990 edition of his book was *Intelligence Running Wild*, which the publisher, HarperCollins, did not approve, suggesting instead *The Seduction of Madness*. He did not like this choice and claimed that he “was always embarrassed by TSM [*The Seduction of Madness*]—I even like the German title better: The Temptation to Madness”.^4^ It seemed that the marketing directors of HaperCollins were convinced that a book with the word “intelligence” in its title would not sell well.^5^ Ironically, also Podvoll's working title for the 2003 edition of his book, *Islands of Clarity*, was not accepted by Shambhala Publications, which proposed instead *Recovering Sanity*.^6^

The book was divided into two parts, “Parables of Madness” and “The Means of Recovery”. The first part contained a few first‐hand accounts of people who experienced psychosis or extreme mental states and carefully documented their experiences. This included the British army officer John Thomas Perceval, John Custance (the author of *Wisdom, Madness and Folly: The Philosophy of a Lunatic*), the British business man and amateur sailor Donald Crowhurst, the Belgian‐French poet and writer Henri Michaux (according to Podvoll, “one of the most sensitive explorers of psychotic states of mind”; Podvoll 2003, 91), and others. The second part focused on reflections around developing clinical skills and constructing healing communities such as Windhorse. The book received the attention of a few eminent readers. Oliver Sacks, for example, considered it “a radical reconsideration of the psychotic mind and its never‐lost potential for ‘islands of clarity’ and self‐recovery”, as well as “an eloquent, and phenomenologically fascinating, meditation on the structure of mind”. The reading of the book (ed. 2003) excited him to such an extent that he decided to write to Podvoll and arrange a visit to Windhorse: “I was so ‘turned on’ by your book that I contacted the Mass. ‘branch’ of WINDHORSE, and have arranged to go up there for a short visit on December 3. But you are the fons et origo of it all—and I want to see you, see the original WINDHORSE in Boulder”.^7^ Other intellectuals, such as Irvin Yalom and Daniel Goleman, praised *Recovering Sanity*. The American writer and broadcaster Studs Terkel invited Podvoll for a radio interview on his daily radio programme in Chicago.^8^ Despite these public recognitions, the author was dissatisfied with the general reception of his book and complained about it several times in his correspondence. In July 1991, he wrote: “Book was published and just reviewed in England: they called it dangerous, and would lead people astray. Finally, someone understands!”^9^

One of the pivotal themes of *Recovering Sanity* was the emphasis on a person's potential for clarity and “wakefulness”. Even in psychotic states, the author recognised the possibility of discovering *lucida intervalla* (“islands of clarity”), that is, moments of sanity and recovery. To foster this potential, a radical “shift of allegiance” between patient and psychotherapist was necessary. This change of perspective was elaborated in an article entitled “The History of Sanity in Contemplative Psychotherapy”, originally published in 1983 in the Naropa *Journal* and later reprinted in the revised edition of *Recovering Sanity*. The article suggested that two histories existed in every patient. The “history of neurosis”, which was “culturally reinforced by almost all therapeutic belief systems”, consisted of “pain, discouragement, missed opportunities, the continual accumulation of unfulfilled hopes and the consequences of unrealised actions in relationships” (Ibid., 354). It was filled with fear, guilt, aggression, and a war‐like mental disposition. Differently, there was also another history at work in every individual: a “history of sanity”, much more “fleeting and delicate”, somehow clothed by the first one and frequently forgotten in the struggle of mental suffering, yet subtly present, at a closer look. The common example of a patient's state of manic excitement was presented. Words, ideas, associations, memories, plans followed one after another, in a hectic and oppressive flight, until a “spontaneous gap” occurred in the patient's thought process (“Where was I?”). As if one had “lost the plot” and encountered the natural sensation that “one has gone too far in the elaboration of a daydream: a sudden awakening followed by a struggle about which way to go” (Ibid., 355). Similar to a flickering flame, in this unexpected moment of doubt and hesitation resided the chance to begin to acknowledge a different storyline. According to the author, the “history of sanity” was dependent on the courage and curiosity to develop a sense of appreciation and trust towards oneself and others; it was made of “wakefulness, dignity and patience” (Ibid., 356). Thus, a shift in attention was required to illuminate an individual's history of sanity. This idea constituted an implicit challenge to classic psychoanalytic theory, which often framed a patient's “unconscious” in terms of repressed drives and resistances. In contrast, contemplative psychotherapists—closer in this respect to Jungian psychology—viewed the “unconscious” as a site of latent clarity and resilience. This reframing did not reject the Freudian model altogether, but invited rebalancing, by prioritising the recognition of mental clarity as much as analysis of psychic conflict. However, a shift of this kind could not happen, if a practitioner had not experienced it first in him or herself:

The question arises of how to relate the history of sanity, ‘what to look for.’ But the issue is not really what the therapist searches for or hunts; rather it is what the therapist recognizes. There are certain signs and landmark events that characterize wakefulness and sanity in another's life, but they can hardly be noticed until one first experiences and identifies them in oneself. […] Only by studying the nature of our own minds and examining the experience of wakefulness in our own lives can we recognize and appreciate it in others.

(Podvoll 2003, 356)

Accordingly, the contemplative psychotherapist had two tasks: (1) to cultivate their own sense of clarity and wakefulness through sitting meditation practice and (2) to support and enrich a similar development in the patient, by learning to acknowledge the patient's “marks of sanity” (Ibid., 357). A typical situation in which this occurred was when a patient stumbled upon the familiar, unsettling sensation expressed by the words “I don't know who I am any more. I used to know, but now I don't”. Podvoll described this occurrence as a fascinating and mercurial moment in a person's life:

When we try to find out who this self really is or was, there usually occurs an immediate confusion, followed by a series of confabulations. Then the patient makes tenuous attempts to construct a cohesive story that describes the nature of self. Nietzsche called this the major obsession of Western man. A feeling of uncertainty, of cloudiness and doubts, undercuts each attempt to materialise a consistent sense of identity*.*

(Ibid., 360)

He argued that this sense of abrupt confusion about oneself had the power to be as profoundly threatening and as liable to lead to madness. Thus, he referred to psychosis as a desire to “transcend the self”, based on the “hope of arising fresh and purified from an abandoned and disfigured self” (Ibid., 360–361). Nevertheless, the same confusion about one's sense of self, if welcomed in an “environment of clarity” (contemplative psychotherapy or therapeutic households such as Windhorse) could lead to a completely different discovery: the clarity and awareness of being a human being on a deeper level than that illusory sense of self so longed for in an imaginary past or future. Podvoll was not naive about the complexity and length of this path, but seemed to strongly believe in the possibility of investing in the history of sanity, even in those extreme conditions that would not seem to allow it. In an unpublished letter, Bleuler praised those efforts as follows:

Dear Doctor Podvoll,

The treatment of schizophrenic patients and the care for them you have described is outstanding. I asked myself immediately in what way it corresponds to the conception of my father and myself—and to our practical work. In regard to your conception it is in many respects more systematic and clearer than ours but the correspondence in regard to the main ideas is overwhelming. In regard to the correspondence of our practical work with schizophrenics I have to confess painful differences: during my life time and even more during my father's life time we had not the possibilities to form teams of helpers—there was by far not enough money to support them. […]

May I mention some particular points in regard to which I found your presentation particularly excellent: the importance of the ‘history of sanity’ is rarely mentioned in the psychotherapeutic literature. As far as I know you are the first who describes it in such a convincing way. It plays also a great role in my psychiatric work—the need to be free from the prejudice that a person who has become insane will always be so, is extremely urgent and you are formulating it very well.^10^

Upon completion of *Recovering Sanity*, Podvoll began to increasingly focus on his meditation practice. Following Trungpa's instructions, *Shamatha* (generally translated as “tranquillity” or “calm abiding”) and *Vipashyana* (“insight” or “wisdom”) meditation, as well as a combination of the two, were the main contemplative practices that were taught at Naropa. Alongside, the practices of *Ngöndro* (lit. “what goes before”), usually translated as foundation practices on the nature of reality, and *Tonglen* meditation (*Tong*, “sending” and *len*, “receiving”; also known as the practice of “exchanging self with others”), which focused on cultivating compassion, were also taught. Around 1980, Trungpa offered a 3‐month long training into a few *Vajrayāna* teachings (lit. “the way of the diamond” or the “diamond vehicle”), that is, an esoteric and lineage‐based Buddhist tradition which evolved in various forms across Asia, but specifically informed Tibetan Buddhism. Following that, alongside other practices, Trungpa's formulation of the Shambhala path informed not only Podvoll's practice, but Naropa and Windhorse communities. As framed by Trungpa, Shambhala teachings were a fully secular way of integrating meditation into every aspect of one's daily life, towards the realisation of an “enlightened society” (Trungpa 1984). Podvoll and Fortuna began to think of the Windhorse activities as an expression of the Shambhala path, which included, among others, creating a “sacred place” at home, in one's household. Thus, until the late 1980s, Podvoll's involvement with meditation developed in close connection to Trungpa's teachings. His dedication to the Tibetan master was unmistakable. This reflects a typical trait of Tibetan Buddhism, in which the guru (Lama) is venerated as a manifestation of the Buddha who acts as an indispensable guide on the spiritual path. Such devotion involves prayers, offerings, prostrations, and a wholehearted willingness to trust the guru's teachings. Podvoll's reverence for Trungpa should thus be understood in relation to this particular aspect of Tibetan Buddhism. It was poignantly expressed in a short piece entitled “Lotus Feet”, which he once wished to be his official obituary, whenever the time came.^11^ “Lotus Feet” recounted Podvoll's discomfort while carrying out a ritual task which Trungpa assigned him (putting his socks and shoes on him) and contained, in his view, the essence of the way Trungpa taught:

Kneeling in front of his naked feet I was struck by how different they were from ordinary feet. They were quite delicately shaped with high arches like a dancer's feet. The skin was clear but had a surprisingly blue‐greenish color, almost translucent. There were a couple of small surgical scars on the top of the left foot, which I imagined were to relieve the contractures caused by his partial paralysis. […] I wanted to do something but I kept thinking how anyone in ancient India, from royalty to beggars, would have seized a once in a lifetime opportunity to place the naked feet of a siddha to the top of his head. Just then I felt his warmth as he leaned over toward my face, looked over the top of his glasses, and in a half‐whisper said, ‘Do you have any children?’

That did it. I was in gear. I telescoped the socks, then took each foot in turn by the ankle, rested it on my knee and in a fluid motion put on each sock while smoothing out any wrinkles to avoid pressure points. I did it as easily as I had put on the socks and shoes of my daughter, getting her ready for kindergarten while she sat eating her cereal while gazing at the pictures on the cornflakes box.

(Podvoll 2022, 83)

By 1990, Podvoll was familiar with a variety of meditation practices. In the summer of the same year, at 54, he and Candace left Colorado and traveled to India, living for some time in an ashram in Tamil Nadu, Southern India. Over the next few months, they left the ashram and went on search of teachers who could give them spiritual direction. Finally, in January 1991, following the guidance of Lama Gendun Rinpoche, he decided to travel back to Europe and enter a traditional Tibetan Buddhist 3‐year retreat in Le Bost, France. Although he and Candace initially thought of doing the retreat together as a couple, eventually they were separated in two groups, one of women and the other of men. In July 1994, he took full ordination. Lama Gendun gave him the Tibetan name Lama Sampa Mingyur (meaning “Immovable Intention for Enlightenment”). In a letter to Fortuna, he commented: “There is of course no way to understand all this—I certainly don't, but merely to take one step after the other as wakefully as possible (= ‘May my mind become one with the Dharma’)”.^12^ Starting from the autumn of 1994, he decided to enter life retreat, committing himself to live in a small group retreat of men for the rest of his life. He sold his possessions and said goodbye to his family and friends, although he maintained a fairly regular correspondence with his closest acquaintances, especially with Fortuna, with whom he frequently talked about the developments of Windhorse. Interestingly, although his main occupations in the following years centred around Buddhist practices, he did not seem to lose interest in intellectual projects. In fact, between 1997 and 1998, he wrote the handwritten copy of the essay “Psychotherapy”, which was later published in *Recovering Sanity*. Written at the core of his life retreat, this essay contained a few reflections around psychotherapeutic technique. In May 2001, 7 years into life retreat, he made an unexpected discovery that brought a further revolution to his life. Around the time of his death in 1997, Lama Gendun had left instructions for Podvoll/Mingyur and others to give up lifelong vows and leave their retreat after 12 years. This meant that in 2001 he had ‘only’ another 5 years of retreat ahead of him. This left him in a state of great uncertainty about what to do and even what name to go by (Dr Edward Podvoll? Lama Mingyur?). His letters from this period reveal that he felt greatly confused by the news, but also elated at the prospect of seeing his friends in Boulder again. In 2002, he decided to leave retreat as he began to feel sick. Upon medical examination in France, he was diagnosed with cancer. He returned to Boulder to receive treatment and reconnect with his friends and colleagues. Under these circumstances, a Windhorse team was created for him, using the same model of care that Podvoll himself had helped to create many years earlier, in a full‐circle moment. He seemed to be both amused and touched by experiencing Windhorse as a client. In one of his last letters, on February 17, 2003, he wrote: “How great it is to have a Windhorse team! It is the big secret. What a gift it is to be cared for and loved. It has made all the difference”.^13^ During the last year of his life, aged 67, he gave teachings on death and the experience of dying, until passing in his home in December 2003.

## 4 Conclusion

This paper aimed to (1) introduce a few key themes in Podvoll's life and work and (2) situate his contributions within the broader historical and conceptual context of the dialogue between Buddhism and Western psychology. In doing so, the paper explored the foundation of a “contemplative psychotherapy” model at Naropa University as one of the earliest examples of the dialogue between Buddhist philosophy and Western clinical traditions in the 20th century. This encounter was mutually transformative, giving rise to new ways of understanding mental suffering and healing processes. As Western psychologies emerged and diversified in the nineteenth and twentieth centuries, they provided frameworks through which Buddhist theories of mind and meditation could be reinterpreted, often as an “ancient psychology” compatible with modern scientific discourse. At the same time, Buddhist teachings were not merely assimilated into existing paradigms; they actively reshaped psychological thought, inviting new forms of self‐inquiry and therapeutic intervention rooted in contemplative experience.

This reciprocal process, often described as the “psychologisation” of Buddhism, was not merely conceptual but institutional and clinical. It found expression in the development of new disciplines, training programs, journals, and therapeutic communities, of which the “contemplative psychotherapy” project at Naropa University is an illustrative case. In this context, Podvoll's work offers a window into the broader historical effort to create a dialogue between Buddhist meditative traditions and Western psychoanalytic and psychiatric thought. The Naropa‐based model was among the earliest to formalise this East–West integration in an academic and therapeutic setting, although its emphasis on subjective experience and meditation‐based insight has also raised questions about the clinical generalisability of such approaches outside committed spiritual contexts. It anticipated many of the themes that would become central to later movements, including the mindfulness‐based interventions that have come to dominate contemporary psychotherapy. Yet contemplative psychotherapy, at least in its intentions, differed in its stance: rather than abstracting mindfulness from its spiritual roots, it aimed to hold Buddhist insight and Western psychology in critical conversation. This orientation shaped the publication aims of *The Journal of Contemplative Psychotherapy*, the founding of the Windhorse communities, and a generation of clinicians working towards attending to both psychological needs and meditative experiences.

Projects like Windhorse echoed contemporaneous experiments in therapeutic community psychiatry, such as those initiated by R.D. Laing, Maxwell Jones, and Manfred Bleuler, but whereas other models often struggled to survive beyond their founders, Windhorse appeared to establish an enduring framework that continues to develop today. In this broader view, contemplative psychotherapy reflected a historical moment when Western therapists began to question the limits of their own epistemologies and turned toward Buddhist traditions less as exotic alternatives than integrative hermeneutics. As the dialogue of meditation and psychotherapy continues to evolve, Podvoll's work and the contemplative psychotherapy project represent a relevant antecedent in the modern genealogy of the intersection of Buddhism and psychology.

## Conflicts of Interest

The author declares no conflicts of interest.

## Data Availability

The author has nothing to report.

## Endnotes

1 Edward Podvoll to Jeffrey Fortuna. July 9, 1997. WLA: 186.

2 Edward Podvoll to Jeffrey Fortuna. January 27, 1999. WLA: 243.

3 Edward Podvoll to Jeffrey Fortuna. March 24, 1992. WLA: 79.

4 Edward Podvoll to Jeffrey Fortuna. June 30, 1999. WLA: 269.

5 Edward Podvoll to Jeffrey Fortuna. November 2, 2001. WLA: 409.

6 Jeffrey Fortuna, personal communication to the author.

7 Oliver Sacks to Edward Podvoll. November 17, 2003. WLA: 521.

8 Recorded on September 13, 1990. WLA.

9 Edward Podvoll to Jeffrey and Molly Fortuna. July 10, 1991. WLA: 60.

10 Manfred Bleuler to Edward Podvoll. October 6, 1984. WLA: 3‐4.

11 Edward Podvoll to Jeffrey Fortuna. July 6, 2001. WLA: 389.

12 Edward Podvoll to Jeffrey Fortuna, September 4, 1994. WLA: 126.

13 Edward Podvoll to Caroline. February 17, 2003. WLA: 488.
